# Altered brain network organization in adults with Asperger's syndrome: decreased connectome transitivity and assortativity with increased global efficiency

**DOI:** 10.3389/fpsyt.2023.1223147

**Published:** 2023-08-17

**Authors:** Nooshin Javaheripour, Gerd Wagner, Feliberto de la Cruz, Martin Walter, Gregor R. Szycik, Fabian-Alexander Tietze

**Affiliations:** ^1^Department of Psychiatry and Psychotherapy, Jena University Hospital, Jena, Germany; ^2^Center for Intervention and Research on Adaptive and Maladaptive Brain Circuits Underlying Mental Health (C-I-R-C), Jena, Germany; ^3^Department of Psychosomatic Medicine and Psychotherapy, Jena University Hospital, Jena, Germany; ^4^Clinical Affective Neuroimaging Laboratory (CANLAB), Magdeburg, Germany; ^5^Department of Psychiatry and Psychotherapy, University of Tübingen, Tübingen, Germany; ^6^Leibniz Institute for Neurobiology, Magdeburg, Germany; ^7^Center for Behavioral Brain Sciences, Magdeburg, Germany; ^8^German Center for Mental Health (DZPG), Jena, Germany; ^9^Department of Psychiatry and Psychotherapy, Hannover Medical School, Hannover, Germany; ^10^Department of Psychiatry and Psychotherapy, Jüdisches Krankenhaus Berlin—Berlin Jewish Hospital, Academic Teaching Hospital of the Charité—Universitätsmedizin Berlin, Berlin, Germany

**Keywords:** autism spectrum disorder (ASD), Asperger's syndrome (AS), graph theory, resting-state functional magnetic resonance imaging (rs-fMRI), autism spectrum traits

## Abstract

**Introduction:**

Autism spectrum disorder (ASD) is a neurodevelopmental disorder that persists into adulthood with both social and cognitive disturbances. Asperger's syndrome (AS) was a distinguished subcategory of autism in the DSM-IV-TR defined by specific symptoms including difficulties in social interactions, inflexible thinking patterns, and repetitive behaviour without any delay in language or cognitive development. Studying the functional brain organization of individuals with these specific symptoms may help to better understand Autism spectrum symptoms.

**Methods:**

The aim of this study is therefore to investigate functional connectivity as well as functional network organization characteristics using graph-theory measures of the whole brain in male adults with AS compared to healthy controls (HC) (AS: *n* = 15, age range 21–55 (mean ± sd: 39.5 ± 11.6), HC: *n* = 15, age range 22–57 [mean ± sd: 33.5 ± 8.5]).

**Results:**

No significant differences were found when comparing the region-by-region connectivity at the whole-brain level between the AS group and HC. However, measures of “transitivity,” which reflect local information processing and functional segregation, and “assortativity,” indicating network resilience, were reduced in the AS group compared to HC. On the other hand, global efficiency, which represents the overall effectiveness and speed of information transfer across the entire brain network, was increased in the AS group.

**Discussion:**

Our findings suggest that individuals with AS may have alterations in the organization and functioning of brain networks, which could contribute to the distinctive cognitive and behavioural features associated with this condition. We suggest further research to explore the association between these altered functional patterns in brain networks and specific behavioral traits observed in individuals with AS, which could provide valuable insights into the underlying mechanisms of its symptomatology.

## Introduction

Autism spectrum disorder (ASD) is a neurodevelopmental condition, which has been recognized as a spectrum disorder since its re-evaluation in the fifth edition of the *Diagnostic and Statistical Manual of Mental Disorders* (DSM-5) in 2013 (1). ASD is characterized by a combination of (1) alterations in social interaction, (2) communicative behavior, and (3) repetitive patterns of behaviors and narrowly circumscribed interests. It additionally takes into account the subdivision of autistic individuals in terms of the severity of their impairments and the need for support in everyday life. Referring to the preceding classifications in the text-revised, fourth edition of the *Diagnostic and Statistical Manual of Mental Disorders* (DSM-IV-TR) and the 10th edition of the *International Classification of Diseases* (ICD-10), autism was not only identified with all three core symptoms mentioned above but also included several subcategories including Asperger's syndrome (AS), Rett's syndrome, Kanner's syndrome, childhood disintegrative disorder, and atypical autism (2, 3). Following the DSM-IV-TR, AS comprised all the typical traits of the autistic triad but was typically not associated with delays in speech and cognitive development (2). It additionally included atypia of communicative behavior and special linguistic elements such as differing prosody or rhythm of speech (4). As a member of the group of pervasive development disorders, AS generally persists into adulthood.

Understanding the importance of AS lies in recognizing the unique strengths and challenges associated with it. Individuals with AS often exhibit remarkable focus and attention to detail impacting their fluid and general intelligence (5, 6). These strengths can contribute to their success in various domains, including academic pursuits as well as detailed and highly original problem-solving strategies (6). However, individuals with AS may also face difficulties in social interactions, especially in noisy conditions, which can be attributed to abnormal sensitivity to sensory stimuli, atypical eye contact, and altered speech perception (7–9). Recognizing the significance of Asperger's syndrome is essential for several reasons. First, it promotes a better understanding and acceptance of neurodiversity (10), acknowledging that individuals with AS have unique strengths and abilities that can contribute positively to society. Second, it allows for tailored support and accommodations to address the challenges individuals with AS may face in social interactions and sensory processing. Asperger's syndrome, as a subtype of autism spectrum disorder, holds significance in understanding the diverse range of strengths and challenges experienced by individuals on the autism spectrum. By recognizing the importance of AS and exploring the neural basis of this condition, we can base further research line, which is more adaptive to their unique brain functionality (11, 12).

Non-invasive neuroimaging studies have broadened our knowledge of both structural and functional neural alterations in ASD. The use of resting-state functional magnetic resonance imaging (rs-fMRI) enabled us to study the correspondence between autistic traits and brain functional connectome without any explicit task. In this context, age has been discussed to play an important role in ASD-related disturbances as significant changes in brain connectivity have already been hypothesized in late adolescence and early adulthood (13, 14). In addition, ASD is sometimes recognized very late or not at all in affected individuals, especially in high-functioning individuals, making research into the neural basis of this condition in adulthood particularly important (15). However, multiple studies have shown that altered functional connectivity in ASD is heterogeneous, making it difficult to reach a consensus on the nature and clinical relevance of these changes (16, 17). Despite these inconsistencies, studying functional connectivity at the resting state in individuals with Asperger's syndrome was shown to provide valuable insights into the neural networks associated with this specific subtype of ASD (14). Therefore, further studies are needed to fully elucidate the role of functional connectivity in autism and its subcategories like AS.

Graph theory has been extensively employed to investigate alterations in brain functional networks in both healthy individuals and psychiatric populations (18). It has provided valuable insights into the underlying mechanisms and potential biomarkers of various psychiatric conditions, such as schizophrenia (19), conduct disorder (20), and dissociative experiences (21). In neuroscience, graph theory can provide a framework to analyze the connectivity patterns between different brain regions, treating them as nodes and the connections between them as edges. These nodes and edges are organized into multilevel networks based on their specific functions, such as sensory or higher order cognitive functions. Ultimately, these networks come together to form the global brain network. The interactions between different regions within this network can be measured using graph theory properties, providing a deeper understanding of brain function and dysfunction. These properties include the clustering tendency of nodes within specific local brain networks and the neuronal path lengths between those networks (22). One way to quantify the organization of a complex network is to form the ratio of the “*clustering coefficient*” of networks to the average shortest path length within the vertices of a network, termed small-worldness. The small-worldness reflects the fact that nodes within or between networks can be reached with a few steps via their immediate neighbors, even if located in separate clusters. This feature indicates if the brain network can efficiently integrate simultaneous information of different quality across neural pathways or segregate them into modality-specific modules within a network (23). The average length of neural pathways within a network negatively correlates with the “*global efficiency”* of a network (18). Another metric for efficient brain processing involves the functioning of specialized and segregated clusters. In addition to global efficiency, networks must also consist of local centers with specialized functions, such as processing specific sensory stimuli. This property of networks can be assessed by measuring the “*segregation.”* As a component of segregation, “*transitivity”* refers to the clustering coefficient of brain networks and therefore reflects the interconnectivity of nodes within their direct neighborhood (18). “*Assortativity”* as a further metric of network segregation reflects the vulnerability of brain networks to harmful events or neuropathological processes, such as stroke, neurodegeneration, or traumatic microlesions (24, 25). High *assortativity* originates from robust connectivity between nodes in given networks corresponding with narrowly distributed links between major nodal hubs in those networks and is therefore interpreted as a marker for network resilience.

Previous studies have reported abnormality in the small-world configuration of the brain network in several psychiatric diseases (26–28), which resulted in the broad application of graph theory-based methods to study brain connectome in ASD (13, 26, 29–37). Because of the complex structure of its symptomatology including alterations in social interaction and communication, changes in ASD were discussed to originate from multiple parts of the brain, such as areas related to multimodal language processing or the Theory of Mind (9, 38). Furthermore, it has been suggested that because of this complex structure, changes in brain organization must predominantly occur at a global level rather than in terms of isolated functional connectivity abnormalities between or within particular brain regions (29). Age was again postulated to constitute another important covariate in the expression of ASD symptomatology, especially at critical developmental stages of the brain such as adolescence and early adulthood (13, 32, 39–42). In this context, research by Henry et al. described an initial decrease in global efficiency in childhood and adolescence with ASD, followed by an increase in early adulthood, while segregation of brain networks was found to increase in childhood and then decrease afterward (13). Confusingly, another study found that network segregation and global efficiency in ASD individuals were both diminished compared with healthy controls (39). Following the findings by Henry et al. (13), ASD can be characterized by a more extreme progression of the typically inverted U-shaped increasing curve of integrated connectome topology from childhood/adolescence into adulthood, which appears to be typical for the developing brain at this stage of life (40). From this perspective, ASD in adulthood would be mainly characterized by the presence of higher global integration (lower characteristic path length) as well as lower clustering and network resilience compared with healthy cohorts (41–43). Interestingly, Itahashi et al. (42) also stressed the need for further studies concentrating on changes in network organization from adolescence into adulthood.

Here, we hypothesized that complex network measures in adults with AS might be altered when compared to the control group. Following the existing literature on adult ASD, we expected functional connectivity alteration within sensory-motor regions as well as between frontal and parietal regions (44). We assumed that assortativity and transitivity of brain networks might be reduced in individuals with AS, whereas metrics quantifying the functional integration of the global connectome might be increased. We aimed for the whole-brain approach and its promising recent applications to psychiatric and autistic cohorts (13, 29–37). Measuring functional connectivity between each pair of regions throughout the whole brain as well as investigating graph theory measures were already subject to scientific research on autism and postulated to provide higher accuracy in diagnosing ASD in male adults (45, 46). For quantifying functional network organization, we investigated group differences in graph theory metrics, including functional segregation (transitivity), functional integration (global efficiency), and network resilience (assortativity coefficient) using the Brain Connectivity Toolbox (18).

## Methods

### Participants

Fifteen male participants diagnosed with Asperger's syndrome (AS) based on DSM-IV-TR (47), and fifteen healthy gender- and age-matched (only male) individuals without any psychiatric or neurologic disorders were recruited in this study as the healthy comparison group (HC). All participants were native German speakers. The final sample consisted of 15 male participants with AS with an age range of 21 to 55 (mean ± SD: 39.5 ± 11.6) and 15 male age-matched healthy controls from 22 to 57 (mean ± SD: 33.5 ± 8.5), and the group comparison using a two-sample *t*-test did not show any significantly different regarding age (*t*-value = 1.61, *p*-value = 0.12). The mean verbal IQ as assessed by the MWT-B—“Mehrfachwahl-Wortschatz-Intelligenztest” (48) was 30.20 for participants with AS and 31.43 for healthy controls and was not significantly different between the groups in the two-sample *t*-test (*t*-value = 0.73, *p*-value = 0.47). The mean of the autism spectrum quotient (AQ) for the AS group was 40.33 ± 5.45 (49), indicating a high expression of autistic traits. We explored DSM-IV-TR criteria for AS through a self-developed semi-structured interview (“Diagnostic interview: AS in adulthood”). This interview contained a general section focusing on medical anamnesis (somatic, psychiatric, and social histories, including childhood development) and continued with a special section involving AS that included the following items with respect to childhood and adulthood: social interaction and communication (e.g., friendships with/relationship to/interest in peers, and being a loner and suffering from loneliness); special interests (e.g., spending leisure time and interest in specific objects/topics); stereotypic behavior (e.g., rituals and reaction toward disturbances of rituals); and other characteristics (e.g., clumsiness and sensitivity toward noises/smells/tactile stimuli). It also included items and descriptions of all relevant criteria for the diagnosis of AS as defined in DSM-IV-TR. We confirmed the result of the interview for every AS subject by verifying the threshold value of the AQ. Additionally, eye contact, facial expression, prosody, and “mirroring” of affections and clumsiness during the interview were included in the assessment. The interview was always conducted by the same experienced psychiatrist and had a duration of ~90 min. For the whole duration of the interview, the investigator was blind to the research questions. The diagnosis was completed with information from personal interviews, gained by telephone or in written form, from observers in childhood and/or adulthood (e.g., partners, friends, and parents or siblings), and sometimes from incorporated school reports. Moreover, retrospective data on the development of speech were assessed. All DSM-IV-TR criteria had to be clearly fulfilled to confirm the diagnosis. An additional examination for axis-I co-morbidity was undertaken by using the German version of the Structured Clinical Interview for DSM-IV Axis I Disorders (SCID-I) (50). All probands of the AS group were diagnosed in adulthood and have not received any psychopharmacological treatment.

The present study was approved by the local Ethics Committee of the Hannover Medical School and has been performed following the latest version of the Declaration of Helsinki (51). The participants gave written informed consent before their participation and took part in the study for a small financial compensation for their travel expenses.

### Scanner information

MR images were acquired on a 3-T Siemens Skyra Scanner (Siemens, Erlangen, Germany) equipped with a standard head coil. A total of 640 T2*-weighted volumes of the whole brain were collected in the AC-PC orientation with the following parameters: repetition time (TR) = 2,400 ms, echo time (TE) = 30 ms, flip angle = 80°, and a field of view (FOV) of 192 mm, matrix size = 64 × 64, 30 transversal slices, and voxel resolution of 3 × 3 × 3 mm3 with an interslice gap 0.33 mm. After the fMRI scan, a 3D high-resolution T1-weighted anatomical scan (MPRAGE-sequence, 192 sagittal slices, FOV = 256 mm, voxel resolution: 1 × 1 × 1 mm3, TR = 2.4 s, TE = 4.37 ms, flip angle = 7°) was recorded. The subject's head was fixed during the entire measurement to avoid head movements.

### Image preprocessing

In the first step, DICOM data were converted to NIFTI files and organized in BIDS (Brain Imaging Data Structure) using dcm2bids (version: 2.1.4). Then, fMRIPrep (version:1.5.5) was used for preprocessing of the structural and functional MR data (52, 53), based on Nipype 1.4.0 (54, 55). The complete preprocessing pipeline could be found in the Supplementary material.

In brief, the functional images were slice-time corrected using 3dTshift from AFNI (56) and corrected for head motion. The T1 image was corrected for intensity non-uniformity, and the non-brain tissue was removed. Subsequently, the functional images were then co-registered to the T1w reference using bbregister (FreeSurfer), which implements boundary-based registration (57). Then, T1 images were normalized to the MNI space by applying non-linear registration using Advanced Normalization Tools (ANTs, version: 2.2.0). The Gaussian kernel of 6 mm FWHM (full-width half-maximum) was used for spatial smoothing with mcflirt (FSL 5.0.9) (58).

Head-motion parameters, i.e., transformation matrices, and six corresponding rotation and translation parameters were used to calculate the framewise displacement (FD) as implemented in Nipype (59) (https://fmriprep.readthedocs.io/en/latest/workflows.html).

### Brain parcellation and identifying brain networks

To test our first hypothesis, AS and healthy control groups were compared for differences in functional connectivity at the network level. For this, we used the parcellation method by Schaefer et al. (61), which consists of 400 regions, to extract the time series of the cortical areas. These 400 regions were categorized into seven brain networks based on the classification by Thomas Yeo et al. (60), namely frontoparietal network (FPN), the default mode network (DMN), salience network (SN), limbic network (LN), dorsal attention network (DAN), sensorimotor network (SMN), and visual network (VN). Several studies have replicated the functional networks of this parcellation scheme based on extended resting-state data.

This homogeneous parcellation is composed of all cortical regions based on fMRI scans of 1,489 participants (61). The Fisher-Z transformation was applied for individual correlation coefficient matrices based on 400 parcels. Each value of these matrices is representative of functional connectivity between two nodes. Additionally, we also compared FC at the nodal level from all 400 ROIs (79,800 FCs) between AS patients and healthy controls.

### Statistical models

To compare AS and control groups regarding the FC metrics at the network and nodal level, we used the two-sample *t*-test. To have comparable normalized variables, the dependent variables were inverse normal transformed before the two-sample *t*-test comparisons (62). To correct for multiple comparisons using the Bonferroni method, the alpha (0.05) was divided by 7, the number of comparisons at the network level (*p*-value < 0.007). However, due to a large number of comparisons at the pairwise FC level (79,800 FCs), we used the false discovery rate (FDR) correction to balance 214 between the likelihood of making a type I error and the probability of making a type II error (63).

### Graph theoretical analysis

We computed both the clustering coefficient and transitivity to investigate the network segregation for different network densities. The clustering coefficient quantifies how much nodes in a network tend to cluster together. It considers the connectivity of both high- and low-degree nodes in the network. However, because the clustering coefficient is normalized at the nodal level, it gives low-degree nodes the same weight as high-degree nodes. This means that low-degree nodes are not ignored when computing the clustering coefficient, and their contribution to the overall network structure is considered. Therefore, networks with higher clustering coefficients might reflect locally efficient networks and suffer from fewer connected nodes. Meanwhile, transitivity is normalized collectively and less influenced by nodes with fewer connections (18). To measure the integration of the brain organization, we have chosen global efficiency as this graph feature is less influenced by long paths and it might be more reliable to show the information flow of a network (64). We also calculated the assortativity coefficient to examine the resilience of a network against “attacks” (65). The assortativity coefficient is a correlation coefficient between the degrees of all nodes on two ends of a link (66).

The correlation matrices of time series from 400 nodes based on the Schaefer parcellation scheme were calculated for each subject (61). These adjacency matrices were used to compute the graph theory features.

Graph theoretical measures were investigated at different network density thresholds, ensuring that all constructed networks had an equal number of edges for a given density (67). We used network densities ranging from 10 to 34% at intervals of 1%. Brain functional networks of this range show small-world properties, as described previously by Zhang et al. (68).

To perform group comparisons of graph theoretical measures, we employed a bootstrapping approach. First, we generated 1,000 bootstrap samples (with replacement) for each group and computed an average connectivity matrix for each sample. Second, we computed the clustering coefficient, transitivity, global efficiency, and assortativity from the average connectivity matrix of each bootstrap sample after thresholding and binarizing over the range of density thresholds mentioned above. Then, we calculated the area under the curve (AUC) for each graph feature. Finally, the comparisons between groups of AUC were made using a two-sample *t*-test and corrected for multiple comparisons using Bonferroni correction.

## Results

### Network-level and nodal-level functional connectivity

To compare the overall functional connectivity within each of the seven brain networks, we averaged the connections within regions of each defined functional network (DMN, FPN, SN, DAN, SMN, VN, and LN). There were no significant differences between the AS and control groups by comparing averaged FCs within seven main cortical networks.

We also compared all 79,800 FC between 400 regions. However, our analysis did not indicate any significant differences between the AS and control groups at the FDR-corrected *p*-level.

### Graph theory properties

**Figure 1:**
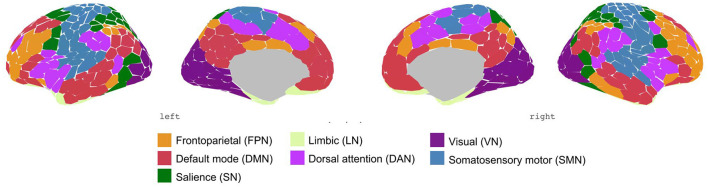
Schaefer parcellation scheme with 400-area categorized in seven networks by Thomas Yeo et al. (60).

**Figure 2:**
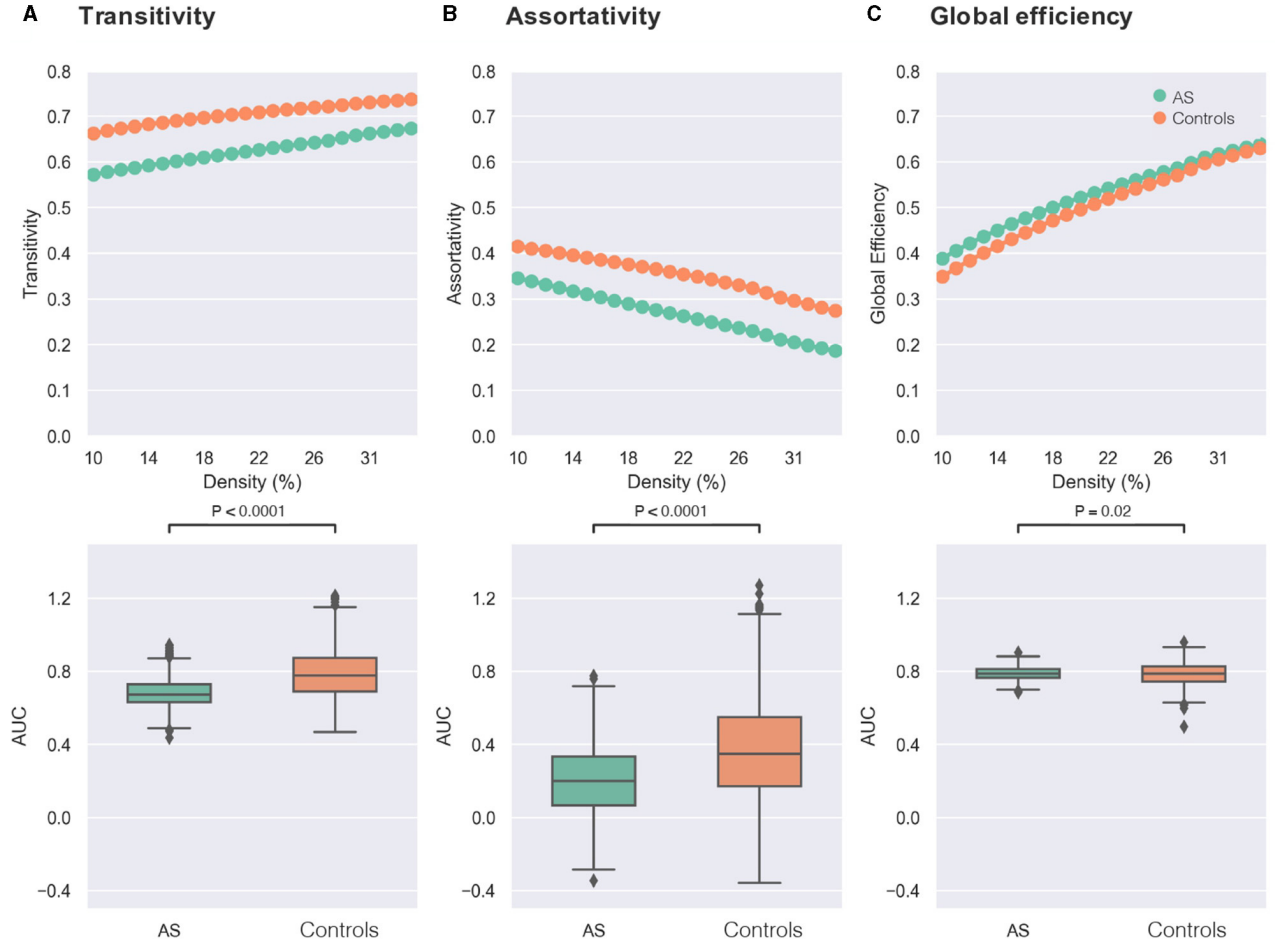
Comparisons of graph theory metrics between individuals with Asperger's syndrome (AS) and healthy controls. In the first row, the X-axis of each plot represents the density threshold ranging from 10 to 34%, and the Y-axis displays the values of graph theory measure, including transitivity **(A)**, assortativity **(B)**, and global efficiency **(C)**. The Asperger's syndrome group (AS) is denoted by green, while the control group is indicated by orange. The second row shows boxplots of the area under the curve (AUC) for each graph measure [transitivity in **(A)**, assortativity in **(B)**, and global efficiency in **(C)**], which are compared between the two groups of AS (green) and controls (orange). The reported *p*-values were corrected for multiple comparisons using the Bonferroni approach (0.05/3).

As presented in Figure 1, significant differences between groups were found in both small-world metrics and the assortativity across several network densities. Regarding transitivity as an indicator of brain network segregation, AS group showed significantly lower segregation than the control group (Figure 2A: AS < controls, *t*-value = −21.20, *p*-value < 0.0001, Bonferroni-corrected). Significant differences in the clustering coefficient confirmed the finding of lower segregation in the AS group (AS < controls, *t*-value = −29.85, *p*-value < 0.0001, Bonferroni-corrected). The AS group showed significantly lower network assortativity compared with healthy controls (Figure 2B: AS < controls, *t*-value = −15.19, *p*-value < 0.0001, Bonferroni-corrected).

Furthermore, the AS group exhibited higher network integration compared with the control group as revealed by higher global efficiency (Figure 2C: AS > controls, *t*-value = 2.33, *p*-value = 0.02, Bonferroni-corrected).

## Discussion

In the present study, we investigated differences in pairwise FC at the whole-brain level as well as in the whole-brain functional organization in individuals with Asperger's syndrome (AS) and HCs. Our study revealed that individuals with AS show decreased network assortativity and transitivity compared with the HC group pointing out reduced activity in local information processing within specified brain regions and lower network resilience in AS. The global efficiency of whole-brain networks was significantly higher in participants with AS compared with HCs, which on the other hand reveals smaller shortest path lengths between distant nodes in AS. Comparing the averaged functional connectivity (FC) within the seven main functional brain networks, we did not find any significant differences between AS and HCs.

Previous studies on brain organization in AS have shown several inconsistencies and are partially competing with the current findings. Our finding of no difference in whole-brain FC in AS individuals compared with the control group is in line with a previous study by Tyszka et al. (46), which found no significant differences in whole-brain FC in adults with high-functioning autism (HFA) in comparison with HCs. However, the aforementioned study considered that mainly individuals in adolescence and with low functional abilities may exhibit FC alterations. Further studies testing this hypothesis demonstrated hyper- and hypoconnectivity in whole-brain analyses of ASD (14, 69, 70). In the case of AS, hyperconnectivity of subcortical and primary sensory networks was found to be associated with disturbed sensory perception in adults with AS (71). Social impairments, one of the core symptoms of AS, were also correlated with hypoconnectivity in insular regions in adolescents with AS (72). Our study provides evidence to support the hypothesis that in adulthood, the brain functional connectivity of individuals with AS might be similar to the functional connectivity of typically developing individuals. This convergence of functional connectivity has already been observed in other brain functions such as sensory processing, which is essential for understanding autistic symptomatology (73). Inconsistencies in previous findings might be also related to high sample heterogeneity regarding age, sex, and severity of symptoms (14, 74). Here, despite the relatively small sample size, our study can contribute important evidence, as our sample contained narrowly defined subjects with AS, exclusively male sex, and high cognitive functioning.

On the other hand, inconsistencies in studies of FC in AS samples could be also related to the complexity of AS symptomatology, which might affect the network organization of the brain. Keeping in mind, that the already mentioned core symptomatology of ASD most likely depends on the interplay of various, also sensory-related, brain networks (9, 38), we argue that the complexity of AS symptomatology could be better explained by relating them to alterations in the whole-brain connectome level analyzed by means of graph theory. Consequently, our results revealed that brain network transitivity and assortativity are globally reduced in the AS cohort, while global integration is increased. This could be interpreted as a shift toward a less efficient and therefore more randomized network configuration in adult AS, which has already been observed in previous connectome studies with other psychiatric cohorts (28). This hypothesis is furthermore in line with studies proving that lower path lengths typically occur in more randomized networks (75, 76). In further consistency with our findings, increased *global efficiency* was found for children with ASD in comparison with matched subjects with developmental delay (30). Moreover, increases in global integration in ASD were reported in studies with ASD samples in childhood and adolescence, showing that global efficiency in male subjects with ASD increased from adolescence into early adulthood, whereas transitivity of brain networks diminished in opposition to this (13).

Partially contradicting our findings are results reporting a decrease in clustering coefficients at the local network level in a sample with ASD individuals in childhood and adolescence (e.g., DMN) (41, 75), at least one of which was based on a highly heterogeneous sample in terms of subjects' verbal IQ, symptom severity, and global functioning (41). Interestingly, segregation deficits in children with ASD have been described for particular brain regions involved in social interaction (77) or emotional face processing (78). Here, we speculate that our finding of no significant alterations at the local network level in adult AS can be explained by the fact that clinically better-performing adults with AS or HFA might only dispose of significant deficits at the global level, whereas deficits in local network connectivity might be associated with earlier stages of brain development in ASD (13, 79). Moreover, our study is in partial contradiction with results that have postulated that significant parameters in ASD can be expected at the local level for the introduction of screening measures (34). However, this study included a sample with a relatively young subject age [age: 20.49 ± 6.16 (14–42)] and may therefore not adequately depict the neural constellation of the adults with AS or HFA.

The findings of our study, therefore, provide further evidence for a characteristic brain network configuration in adults with ASD, which has already been characterized in previous studies by increased global integration and lower clustering coefficients (41–43, 75). However, it is not possible to specify the exact symptoms that can be explained by the present findings as the study did not directly investigate the relationship between network configurations and specific symptoms. The identified network configuration of increased global integration and lower clustering coefficients in AS group may nevertheless provide insights into the underlying neural mechanisms that contribute to this specific subgroup of ASD, characterized by alterations in social communication, repetitive behaviors, and restricted interests without any delay in verbal or cognitive development (2, 3). Further research is needed to establish the relationship between network configurations and specific symptoms of ASD.

### Limitations

In the first line, the sample size is small and diagnostics for Asperger's syndrome are already obsolete. The results of studies only deliver information on a subset of the current ASD population (high-functioning autism/Asperger's syndrome). Another important consideration is the potential impact of gender on brain network organization in ASD and to control the effect of gender, our study only included male participants, which has also been discussed as an independent factor in the context of altered brain functionality in autism (13, 14, 80–82). Additionally, we did not correlate the severity of the AS symptomatology to other clinical scores than the AQ. Therefore, the limitations of our investigation may potentially be the cause of the unexpected results in our study, such as the non-finding in the FC analyses.

## Conclusion

In conclusion, this study showed that global FC in adult AS was significantly different from adult HC. As adults exhibited increased global integration and decreased network transitivity and assortativity. Current findings indicated more randomized brain organization in adult AS compared to HCs. We recommend two areas for future research. First, comparing brain organization measures in different groups of individuals stratified by clusters of ASD symptoms, gender, age, and severity of functional impairments could deepen our understanding of the symptomology of ASD and related disturbances in brain functional organization. Second, longitudinal studies on high-functioning individuals with ASD could help map changes in brain functional organization alongside changes in symptoms from childhood to adulthood.

## Data availability statement

The raw data supporting the conclusions of this article will be made available by the authors, without undue reservation.

## Ethics statement

The present study was approved by the Local Ethics Committee of the Hannover Medical School. The participants gave written informed consent before their participation and took part in the study.

## Author contributions

F-AT and GS participated in the coordination of the study and performed the measurement. NJ, GW, and F-AT conceptualized the topic, scope of the research, and wrote the original draft of the manuscript. NJ and FC performed the data analysis. All authors reviewed the original draft and read and agreed to the published version of the manuscript.
